# A de novo missense variant in *GABRA4* alters receptor function in an epileptic and neurodevelopmental phenotype

**DOI:** 10.1111/epi.17188

**Published:** 2022-02-12

**Authors:** Florian D. Vogel, Martin Krenn, Dominik S. Westphal, Elisabeth Graf, Matias Wagner, Steffen Leiz, Filip Koniuszewski, Maximilian Augé‐Stock, Georg Kramer, Petra Scholze, Margot Ernst

**Affiliations:** ^1^ Department of Pathobiology of the Nervous System Center for Brain Research Medical University Vienna Vienna Austria; ^2^ Department of Neurology Medical University of Vienna Vienna Austria; ^3^ Institute of Human Genetics School of Medicine Klinikum rechts der Isar Technical University of Munich Munich Germany; ^4^ Department of Internal Medicine I School of Medicine Klinikum rechts der Isar Technical University of Munich Munich Germany; ^5^ Institute of Neurogenomics Helmholtz Zentrum München Neuherberg Germany; ^6^ Department of Pediatrics Dr. von Hauner Children's Hospital LMU University Hospital Munich Germany; ^7^ Division of Pediatric Neurology LMU Center for Development and Children with Medical Complexity Ludwig‐Maximilians‐University Munich Munich Germany; ^8^ Divison of Neuropediatrics Klinikum Dritter Orden Munich Germany

**Keywords:** Drug‐resistant epilepsy, early‐onset epilepsy, GABA_A_ receptors, neurosteroid, tonic inhibition, trio exome sequencing

## Abstract

Variants in γ‐aminobutyric acid A (GABA_A_) receptor genes cause different forms of epilepsy and neurodevelopmental disorders. To date, *GABRA4*, encoding the α4‐subunit, has not been associated with a monogenic condition. However, preclinical evidence points toward seizure susceptibility. Here, we report a de novo missense variant in *GABRA4* (c.899C>T, p.Thr300Ile) in an individual with early‐onset drug‐resistant epilepsy and neurodevelopmental abnormalities. An electrophysiological characterization of the variant, which is located in the pore‐forming domain, shows accelerated desensitization and a lack of seizure‐protective neurosteroid function. In conclusion, our findings strongly suggest an association between de novo variation in *GABRA4* and a neurodevelopmental disorder with epilepsy.

## 1 INTRODUCTION

Genetic variants in a subset of genes encoding γ‐aminobutyric acid A (GABA_A_) receptor subunits have already been associated with a wide spectrum of monogenic epilepsies, ranging from self‐limiting febrile seizures to severe developmental and epileptic encephalopathies (DEEs).^1^ Apart from these single‐gene etiologies, an overall mutational burden in several of the 19 GABA_A_ receptor subunit–encoding genes was found to contribute to common focal and generalized epilepsies in a polygenic manner.^2^ Hence, GABA_A_ receptor dysfunction represents one of the most relevant and best‐understood mechanisms underlying genetic epilepsies. Owing to genomic‐sequencing approaches, the number of identified monogenic defects is still growing at a rapid pace.^3^

*GABRA4*, encoding the extrasynaptically located α4‐subunit of the GABA_A_ receptor, has not been associated with genetic epilepsy so far. However, preclinical evidence points toward a pathogenic role in autism‐spectrum disorder and alterations of seizure susceptibility.^4^, ^5^ α4‐subunit‐containing GABA_A_ receptors contribute to tonic inhibition mediated by extrasynaptic receptors, which have been associated with seizures and seizure‐protective effects of neurosteroids.^6^ In addition, *GABRD*, which encodes the extrasynaptic δ‐subunit, is considered as an epilepsy susceptibility gene.^7^, ^8^ Taken together, converging evidence suggests *GABRA4* as a candidate gene for epilepsy.

Here, we report a novel de novo missense variant in *GABRA4* in an individual with early‐onset epilepsy and various neurodevelopmental abnormalities. A comprehensive functional characterization of the detected variant demonstrates a molecular change‐of‐function, thus providing first evidence for an association between variants in *GABRA4* and a neurodevelopmental disorder with epilepsy.

## 2 MATERIAL AND METHODS

For full methods see Supplementary Methods (SM), in brief:

### 2.1 Probands and samples

Written informed consent was obtained for the collection and storage of clinical and imaging data and blood samples and for publication. The study was conducted in agreement with the Declaration of Helsinki and approved by the local ethics committee.

### 2.2 Genetic analyses

Exome sequencing (ES) was performed as reported previously.^9^ For this study, a case‐parent trio was sequenced using *SureSelect Human All Exon Kit* 60 Mb, V6 (Agilent, Santa Clara, California, USA) for exome enrichment. Libraries were sequenced on an *Illumina HiSeq4000* system (Illumina). Reads were aligned to the UCSC human reference assembly (hg19). More than 97% of the exome was covered at least 20‐fold. Average coverage was more than 108‐fold. The criteria of the American College of Medical Genetics and Genomics (ACMG) were applied for variant interpretation (Supplementary Methods).^10^ The reported variant was confirmed by Sanger sequencing from blood and oral mucosa DNA.

### 2.3 Visualization of α4T300I

The crystal structure (PDB ID: 5OSB)^11^ was used for visualization in MAESTRO 12.7.156, in which T300 (T266 in 5OSB) was mutated using the 3D Builder function.

### 2.4 Mutagenesis

To generate the α4T300I mutation, site‐directed mutagenesis was performed with Q5^®^ Site‐Directed Mutagenesis Kit following the protocol. Primers (Supplementary Methods) were purchased from Eurofins Genomics. All constructs were verified via sequencing.

### 2.5 Recombinant expression

Human subunit combinations (α4β2, α4T300Iβ2, α4β3, and α4T300Iβ3) were recombinantly expressed in HEK 293 cells (Supplementary Methods) for western blot analysis (Supplementary Methods). For electrophysiological recordings, messenger RNA (mRNA) of human α4+β2, α4T300I+β2, α4+β3, and α4T300I+β3 were mixed in a 10:1 ratio with a final concentration of 70 ng/µL for microinjection into *Xenopus laevis* oocytes. Oocytes were injected with 46 nL of RNA for a total amount of 3.22 ng. The injected oocytes were incubated at 23°C in (NDE++) for 9–10 days before recording (Supplementary Methods). For similar supporting experiments using rat subunits, see Supplementary Methods and Results.

### 2.6 Functional testing with two electrode voltage clamp in *Xenopus laevis* oocytes

Oocytes were put on a nylon grid and impaled with two microcapillaries filled with 2 M KCl (1–3 MΩ resistance). Oocytes were continuously perfused with NDE buffer or buffer plus GABA/GABA + THDOC (tetrahydrodeoxycorticosterone) (Supplementary Methods). All substances were applied for 30 s. Electrophysiological recordings were performed at room temperature at a holding potential of −60 mV using a Turbo Tec‐03X npi amplifier.

### 2.7 Data analysis

Whole‐cell currents were analyzed as previously described.^12^ The extent of desensitization was calculated as 1−I(_res_)/I(_peak_), where I_res_ is the remaining current when GABA application ends and I_peak_ is the maximal current. THDOC modulation of GABA‐elicited current was defined as (I_GABA+Comp_/I_GABA_), where I_GABA+Comp_ is the current response in the presence of GABA and THDOC and I_GABA_ is the control GABA current. Normalized and averaged traces were generated by normalizing each trace to its peak value. Subsequently, mean ± standard error of the mean (SEM) were determined for the aligned traces (Supplementary Methods). For all statistical analysis presented in the article, ungrouped *t* test with Holm‐Sidak correction was performed.

## 3 RESULTS

### 3.1 Clinical findings

Our female patient was born at term after an uneventful pregnancy. Ten weeks after birth she had pyelonephritis and was admitted to hospital because a fever‐related seizure was suspected. Vesicoureteral reflux causing relapsing episodes of pyelonephritis was treated with endoscopic injection.

Neurodevelopmental abnormalities (predominantly affecting speech) were first noted at 2 years of age. At age 3.5 years, she developed sleep‐related seizures occurring in clusters of several episodes per hour. She suddenly woke up unresponsive with shaking limbs and ballistic movements of the left leg. Prolonged electroencephalography (EEG) recordings showed a right frontal seizure origin with rapid generalization. Later on, interictal frontal epileptic discharges were detected. Magnetic resonance imaging (MRI) of the brain was normal. A comprehensive diagnostic workup including cerebrospinal fluid (CSF) analysis and metabolic and autoimmunological investigations did not reveal a specific etiology.

Given the rapidly increasing seizure frequency, she was admitted to an intensive care unit, requiring a continuous infusion of midazolam (0.16 mg/kg/h). In addition, an antiseizure medication (ASM) regimen including lacosamide (LCM) (5 mg/kg/day) and levetiracetam (LEV) (65 mg/kg/day) was established. Due to agitation, she also received single doses of chloral hydrate and propofol, leading to clinical improvement.

Thereafter, clustering seizures recurred. LEV and LCM were withdrawn due to agitation and replaced by phenobarbital (PB) (6 mg/kg/day), zonisamide (ZNS) (up to 10 mg/kg/day), and valproic acid (VPA) (40 mg/kg/day). Clustering seizures responded temporarily to chloral hydrate. VPA was discontinued because of hepatopathy, which again led to an increased seizure frequency. Add‐on treatment with brivaracetam (BRV) resulted in a significant reduction to one to two nocturnal seizures per night. Lamotrigine (LTG) was phased in, and she was discharged with a combination of ZNS, BRV, and LTG, leading to stabilization. Eventually, LTG was withdrawn and she remained seizure‐free under ZNS and BRV for 2 years, but later experienced a recurrence of sleep‐related hypermotor seizures.

Neuropsychological assessment at 5 years of age indicated dyspraxia and deficits affecting attention, concentration, executive function, and language comprehension. Logopedic examination revealed speech impairment mainly affecting phonological domains. At age 5.5 years, at last follow‐up, she has attended a kindergarten and received special education.

### 3.2 Genetic findings

**FIGURE 1:**
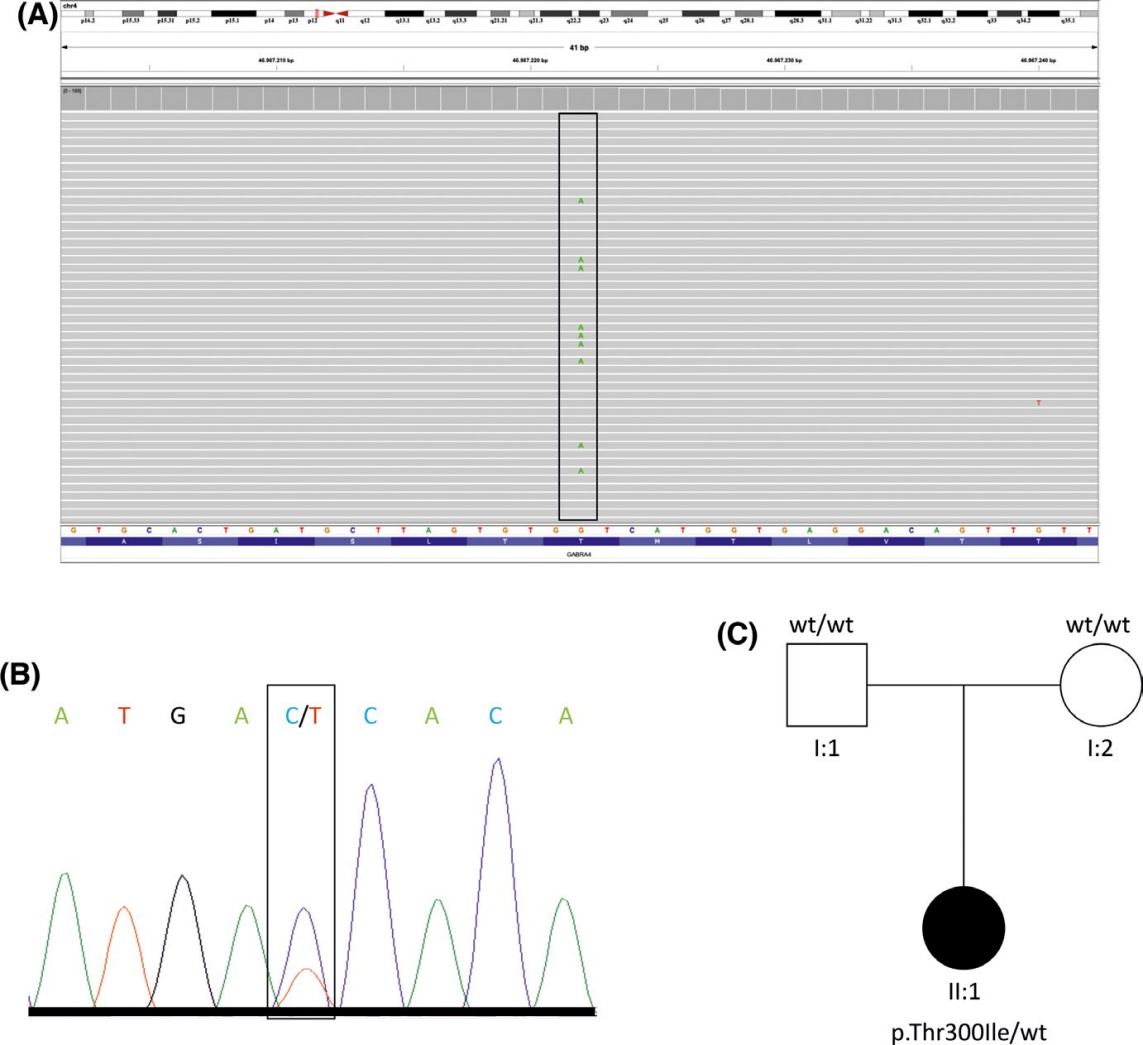
Genetic findings from exome and Sanger sequencing. (A) Integrative genomics viewer (IGV) screenshot displaying exome‐sequencing findings in the index patient with the variant (framed) found in 17% of sequencing reads, indicating mosaicism due to a postzygotic origin of the variant. Each gray lane represents one sequencing read. (B) Electropherogram showing the Sanger‐sequencing results confirming the same variant (framed) at a comparable degree of mosaicism using two different specimens of oral mucosa of the index patient. (C) Schematic pedigree illustrating the two clinically unaffected parents carrying two wild‐type (wt) alleles and the clinically affected daughter carrying the reported de novo variant c.899C>T, p.(Thr300Ile) in *GABRA4* (NM_000809.3)

Trio exome sequencing (ES) identified the variant c.899C>T, p.(Thr300Ile) in *GABRA4* (NM_000809.3), which was absent from gnomAD and our in‐house database (comprising >20 000 exome data sets). Multiple lines of in silico evidence predicted a damaging effect of the variant (PolyPhen‐2: probably damaging [0.998], SIFT: 0, CADD: 28.6). The variant was not detected in both parents, suggesting de novo mutagenesis (Figure 1C). In the index patient, the variant was present in 17% (26/155) of sequencing reads, suggestive of mosaicism (Figure 1A). Subsequent Sanger sequencing from blood (one specimen) and oral mucosa (two specimens) confirmed the variant with a similar degree of mosaicism, confirming a postzygotic origin (Figure 1B).

### 3.3 Molecular characterization

**FIGURE 2:**
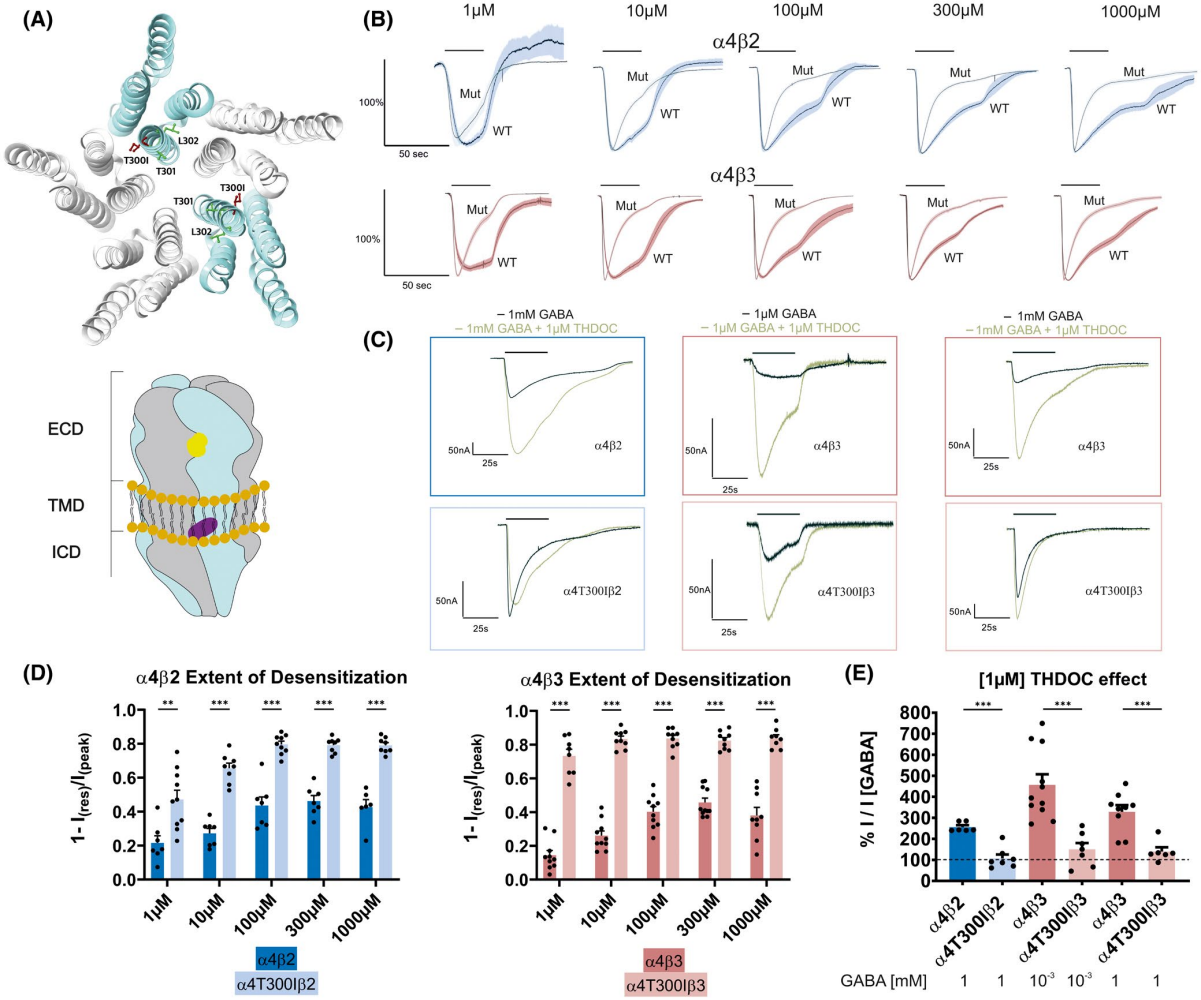
(A) Top: Localization of the mutation (red sticks) in the transmembrane domain (TMD) within the TTL motif (green); α subunits are cyan and β subunits are gray. Bottom: Cartoon side view of an α4β receptor with generally assumed arrangement, showing the extracellular, transmembrane, and intracellular domains (ECD, TMD and ICD) and the localization of the γ‐aminobutyric acid (GABA)–binding site in the ECD (yellow) and the modulatory neurosteroid‐binding site in the TMD (purple). Both sites are localized at the so‐called β+/α− subunit interface. (B) Top: Normalized and averaged GABA‐evoked currents from α4β2 (*n* [1 µM; 10 µM; 100 µM; 300 µM; 1000 µM] = 5); and α4T300Iβ2 (*n* [1 µM; 10 µM; 100 µM; 300 µM; 1000 µM] = 7; 5; 7; 7; 7) subunit combinations. Mean is represented by the black trace; standard error of the mean (SEM) is depictured in dark blue for α4β2 and in light blue for α4T300Iβ2. The bar on the top indicates the approximate application time (30 s). Bottom: Normalized and averaged GABA‐evoked currents from α4β3 (*n* [1 µM; 10 µM; 100 µM; 300 µM; 1000 µM] = 12; 11; 13; 9; 8) and α4T300Iβ3 (*n* [1 µM; 10 µM; 100 µM; 300 µM; 1000 µM] = 9; 10; 10; 10; 7) subunit combinations. Mean is represented by the black trace; SEM is depictured in dark red for α4β3 and in light red for α4T300Iβ3. The bar on the top indicates the approximate application time (30 s). (C) Representative current traces of GABA elicited (black) and THDOC‐modulated (brown) currents. The bar on the top indicates substance application. (D) Quantitative comparison of the extent of desensitization in α4β2 and α4β3 receptors with the respective mutated ones. Data are shown as mean (bars) ± SEM, with each data point representing a biological replicate for the respective subtype and GABA concentration. α4β2: *n* (1 µM; 10 µM; 100 µM; 300 µM; 1000 µM) = 7; 7; 7; 7; 6. α4T300Iβ2: *n* (1 µM; 10 µM; 100 µM; 300 µM; 1000 µM) = 10; 8; 9; 8; 8. α4β3: *n* (1 µM; 10 µM; 100 µM; 300 µM; 1000 µM) = 10; 10; 10; 11; 9. α4T300Iβ3: *n* (1 µM; 10 µM; 100 µM; 300 µM; 1000 µM) = 8; 9; 9; 9; 8. *p* Values: **p < .003; ***p < .001; multiple *t* tests with Holm‐Sidak correction. (E) Quantitative comparison of 1[µM] steroid potentiation of currents elicited by sub‐saturating (1 µM) and saturating (1 mM) GABA concentrations. Dashed line corresponds to reference current (GABA alone). Data are shown as mean (bars) ± SEM, with each data point representing a biological replicate for the respective subtype and GABA concentration. α4β2: *n* (1000 µM) = 6. α4T300Iβ2: *n* (1000 µM) = 7. α4β3: *n* (1 µM; 1000 µM) = 11; 9. α4T300Iβ3: *n* (1 µM; 1000 µM) = 7; 6; ***p < 0.001; multiple *t* tests with Holm‐Sidak correction

Analysis of available atomic resolution structure data indicates that the mutation is localized in the transmembrane domain (TMD), a hot spot for epilepsy‐causing de novo variants in GABA_A_ receptors (Figure 2A).^13^ This variant is localized in a conserved TT(I/L) motif, which is intolerant towards variation. Severe epilepsies having been reported for variants in this motif of β subunits.^13^ To examine differences between wild‐type (WT) and mutated α4‐subunit containing receptors, we expressed α4β2 and α4β3 in comparison with the respective α4T300Iβ(2/3) recombinantly. In HEK293 cells, expression, assembly, and surface trafficking were comparable based on western blot analysis (Figure S1). Electrophysiological examination in *Xenopus laevis* oocytes resulted in comparable GABA‐elicited maximal whole cell amplitudes and dose‐response curves in the pairwise comparisons between the WT and mutated combinations. (Table S1, Figure S2).

Differences between WT‐ and α4T300I‐containing combinations were further explored in detail with respect to GABA and neurosteroid responses. The extent of desensitization was much higher in receptors formed with the α4T300I mutant (Figure 2B,D), indicative of faster channel inactivation. Because human α4‐containing receptors express poorly (Supplementary Methods) and are known to produce lower currents than their rat homologs,^14^ we performed additional experiments with a panel of rat subunits. The desensitization phenotype was well recapitulated in all rat α4T300I‐containing receptors compared to the WT‐containing combinations (Figures S3 and S4).

Endogenous neurosteroids are known for their seizure‐protective function by modulating GABA effects.^15^ In the WT α4β3 receptor, 1 µM of the endogenous neurosteroid tetrahydrodeoxycorticosterone (or THDOC) potentiated submaximal and maximal GABA‐evoked responses. This potentiating effect was markedly reduced in α4T300Iβ3‐expressing cells (Figure 2C,E). Similarly, α4T300Iβ2‐expressing cells are also nearly THDOC insensitive at saturating GABA concentrations (Figure 2C,E).

## 4 DISCUSSION

Here, we report an individual with early‐onset focal epilepsy and neurodevelopmental abnormalities, in whom we identified a *de novo* missense variant in *GABRA4* (c.899C>T, p.Thr300Ile) using trio ES. Our results suggest a postzygotic origin of the variant, leading to somatic mosaicism. Because no brain tissue was analyzed, the degree of mosaicism in epileptogenic brain areas remains unknown. However, the confirmation of the variant in ectodermal tissue (oral mucosa) suggests mutagenesis at an early embryonic stage, thus making brain involvement likely.

A comprehensive functional characterization of the detected variant demonstrated accelerated desensitization and a lack of seizure‐protective neurosteroid function. The α4 subunits contribute chiefly to tonically active receptors in extrasynaptic localizations where they mediate non‐desensitizing low currents elicited by ambient or spillover GABA.^6^ They occur in a vast number of pentameric assemblies, the majority of which contain only α and β subunits,^16^ and thus binding sites for GABA and neurosteroids, but no high‐affinity site for benzodiazepines (Figure 2A; Figure S5). Tonically active receptors contribute to neuronal excitability, and a change of their kinetics from a slow‐ into a fast‐desensitizing type can be expected to destabilize net tonic inhibition. This likely gets further escalated by the loss of neurosteroid sensitivity. In addition to a wide range of neuron types, *GABRA4* is also expressed in astrocytes and microglia.^17^ Yet the role it plays in these cells is still widely unknown.

Molecular phenotypes resembling the electrophysiological findings in our patient, mainly characterized by increased desensitization speed, have been described in other patients with mutations in GABA_A_ receptor genes and epileptic syndromes.^13^, ^18^ We are aware that cellular‐expression systems alone are not sufficient to prove disease causation; however, our electrophysiological findings along with genetic evidence strongly suggest a pathogenic role based on disrupted tonic inhibition.

Although *GABRA4* has not been established as a monogenic epilepsy gene thus far, GABA_A_ receptor–encoding genes in general are among the best‐studied genetic risk factors contributing to epilepsies.^1^ In contrast to our patient, most monogenic GABA_A_ receptor–related epilepsies have been described as generalized epilepsy or DEE phenotypes. Nevertheless, growing evidence points toward both a monogenic and a polygenic role also in focal epilepsies.^9^, ^19^, ^20^

Moreover, we acknowledge that any conclusions that may be drawn from our results are limited, as only one individual and one variant were investigated. Hence, similar findings need to be replicated in additional patients to corroborate *GABRA4* as a monogenic epilepsy gene.

In summary, our findings represent the first genetic and functional evidence for an association between *GABRA4* and a neurodevelopmental disorder with epilepsy. Provided that future replications will reinforce a causal relationship, *GABRA4* may soon be added to the constantly growing list of epilepsy‐related disease genes.

## CONFLICT OF INTEREST

The authors declare that the research was conducted in the absence of any commercial or financial relationships that could be construed as a potential conflict of interest. None of the authors has any conflict of interest to disclose. We confirm that we have read the Journal's position on issues involved in ethical publication and affirm that this report is consistent with those guidelines.

## Supporting information

Supplementary Material

## References

[1] Maljevic S , Møller RS , Reid CA , Pérez‐Palma E , Lal D , May P , et al. Spectrum of GABAA receptor variants in epilepsy. Curr Opin Neurol. 2019;32(2):183–90. 30664068 10.1097/WCO.0000000000000657

[2] May P , Girard S , Harrer M , Bobbili DR , Schubert J , Wolking S , et al. Rare coding variants in genes encoding GABAA receptors in genetic generalised epilepsies: an exome‐based case‐control study. Lancet Neurol. 2018;17(8):699–708. 30033060 10.1016/S1474-4422(18)30215-1

[3] Butler KM , Moody OA , Schuler E , Coryell J , Alexander JJ , Jenkins A , et al. De novo variants in GABRA2 and GABRA5 alter receptor function and contribute to early‐onset epilepsy. Brain. 2018;141(8):2392–405. 29961870 10.1093/brain/awy171 PMC6061692

[4] Fan C , Gao Y , Liang G , Huang L , Wang J , Yang X , et al. Transcriptomics of Gabra4 knockout mice reveals common NMDAR pathways underlying autism, memory, and epilepsy. Mol Autism. 2020;11(1):13. 32033586 10.1186/s13229-020-0318-9 PMC7007694

[5] Roberts DS , Raol YH , Bandyopadhyay S , Lund IV , Budreck EC , Passini MJ , et al. Egr3 stimulation of GABRA4 promoter activity as a mechanism for seizure‐induced up‐regulation of GABAA receptor α4 subunit expression. Proc Natl Acad Sci USA. 2005;102(33):11894–9. 16091474 10.1073/pnas.0501434102 PMC1187961

[6] Brickley SG , Mody I . Extrasynaptic GABA A receptors: their function in the CNS and implications for disease. Neuron. 2012;73(1):23–34. 22243744 10.1016/j.neuron.2011.12.012 PMC3399243

[7] Dibbens LM , Feng HJ , Richards MC , Harkin LA , Hodgson BL , Scott D , et al. GABRD encoding a protein for extra‐ or peri‐ synaptic GABAA receptors is susceptibility locus for generalized epilepsies. Hum Mol Genet. 2004;13(13):1315–9. 15115768 10.1093/hmg/ddh146

[8] Ahring PK , Liao VWY , Gardella E , Johannesen KM , Krey I , Selmer KK , et al. Gain‐of‐function variants in GABRD reveal a novel pathway for neurodevelopmental disorders and epilepsy. Brain. 2021. Epub ahead of print. 10.1093/brain/awab391 PMC9630717 34633442

[9] Krenn M , Wagner M , Hotzy C , Graf E , Weber S , Brunet T , et al. Diagnostic exome sequencing in non‐acquired focal epilepsies highlights a major role of GATOR1 complex genes. J Med Genet. 2020;57(9):624–33. 32086284 10.1136/jmedgenet-2019-106658

[10] Richards S , Aziz N , Bale S , Bick D , Das S , Gastier‐Foster J , et al. Standards and guidelines for the interpretation of sequence variants: a joint consensus recommendation of the American College of Medical Genetics and Genomics and the Association for Molecular Pathology. Genet Med. 2015;17(5):405–24. 25741868 10.1038/gim.2015.30 PMC4544753

[11] Laverty D , Thomas P , Field M , Andersen OJ , Gold MG , Biggin PC , et al. Crystal structures of a GABA A ‐receptor chimera reveal new endogenous neurosteroid‐binding sites. Nat Struct Mol Biol. 2017;24(11):977–85. 28967882 10.1038/nsmb.3477 PMC6853794

[12] Steudle F , Rehman S , Bampali K , Simeone X , Rona Z , Hauser E , et al. A novel de novo variant of GABRA1 causes increased sensitivity for GABA in vitro. Sci Rep. 2020;10(1):2379. 32047208 10.1038/s41598-020-59323-6 PMC7012862

[13] Hernandez CC , Macdonald RL . A structural look at GABAA receptor mutations linked to epilepsy syndromes. Brain Res. 2019;1714:234–47. Elsevier B.V. 30851244 10.1016/j.brainres.2019.03.004

[14] De WM , Rahman M , Johansson IM , Bäckström T . Agonist function of the recombinant α4β 3δ GABAA receptor is dependent on the human and rat variants of the α4‐subunit. Clin Exp Pharmacol Physiol. 2010;37(7):662–9. 20337660 10.1111/j.1440-1681.2010.05374.x

[15] Miziak B , Chrościńska‐Krawczyk M , Czuczwar SJ . Neurosteroids and seizure activity. Front Endocrinol. 2020;11:541802. Frontiers Media S.A. 10.3389/fendo.2020.541802 PMC7561372 33117274

[16] Bencsits E , Ebert V , Tretter V , Sieghart W . A significant part of native γ‐aminobutyric acid(A) receptors containing α4 subunits do not contain γ/or δ subunits. J Biol Chem. 1999;274(28):19613–6. 10391897 10.1074/jbc.274.28.19613

[17] Zhang Y , Sloan SA , Clarke LE , Caneda C , Plaza CA , Blumenthal PD , et al. Purification and characterization of progenitor and mature human astrocytes reveals transcriptional and functional differences with mouse. Neuron. 2016;89(1):37–53. 26687838 10.1016/j.neuron.2015.11.013 PMC4707064

[18] Hernandez CC , Kong W , Hu N , Zhang Y , Shen W , Jackson L , et al. Altered channel conductance states and gating of GABAA receptors by a pore mutation linked to Dravet syndrome. eNeuro. 2017;4(1):ENEURO.0251‐16.2017. 10.1523/ENEURO.0251-16.2017 PMC5301078 28197552

[19] Feng YCA , Howrigan DP , Abbott LE , Tashman K , Cerrato F , Singh T , et al. Ultra‐rare genetic variation in the epilepsies: a whole‐exome sequencing study of 17,606 individuals. Am J Hum Genet. 2019;105(2):267–82. 31327507 10.1016/j.ajhg.2019.05.020 PMC6698801

[20] Reinthaler EM , Dejanovic B , Lal D , Semtner M , Merkler Y , Reinhold A , et al. Rare variants in γ‐aminobutyric acid type A receptor genes in rolandic epilepsy and related syndromes. Ann Neurol. 2015;77(6):972–86. 25726841 10.1002/ana.24395

