# Supplementary material for: A de novo missense variant in GABRA4 alters receptor function in an epileptic and neurodevelopmental phenotype
Source: Epilepsia. 2022 Feb 12;63(4):e35–41. doi: 10.1111/epi.17188 (PMC9304230; doi:10.1111/epi.17188)
Supplement: Supplementary file 1 — Supplementary Material [file EPI-63-e35-s001.doc]

**SUPPLEMENTARY METHODS & FIGURES**

**Exome sequencing and data analysis**

Trio exome sequencing, i.e. including the index patient and both unaffected parents, was performed at the Institute of Human Genetics (Technical University Munich, Germany).

Exomes were enriched in solution with SureSelect Human All Exon Kits 60Mb V6 (Agilent, Santa Clara, California, USA). DNA fragments were sequenced as 100bp paired-end runs on an *Illumina HiSeq4000* system (Illumina, San Diego, California). More than 97% of the exome were covered at least 20-fold. The mean average coverage was 108-fold.

The two following two bioinformatic pipelines were used:

(1) SAMtools: Reads were aligned to the UCSC human reference assembly (hg19) using BWA v.0.5.8. Single-nucleotide variants (SNV) and small insertions and deletions (indel) were detected with SAMtools v0.1.7.

(2) GATK: Reads were aligned to the UCSC human reference assembly (hg19) using BWA v0.5.8. Duplicates were removed using Picard v2.5.0. Indel realignment, base quality realignment (GATK v3.6.0) and variant calling (HaplotypeCaller) was done as recommended by GATK Practices.

Variants were filtered based on the minor allele frequency (MAF), which was estimated using our in-house database (>20,000 exome datasets) and confirmed by gnomAD. Variant prioritization was based on autosomal recessive (MAF <0.1%) and dominant (MAF <0.01%) filters. The standard criteria of the American College of Medical Genetics and Genomics (ACMG) were applied for variant interpretation.

**Recombinant protein expression in HEK-293 cells**

HEK293 cell transfection and membrane preparation**:** Culturing and transfection of Human Embryonic Kidney (HEK) 293 cells with human α4β2 and α4β3 cDNAs (ratio 10:1) with either wild-type α4 or mutant α4T300I was performed as described previously.^1^ Cells were harvested and membranes were prepared as described.^2^

Antibody generation: Antibodies targeted against the cytoplasmic loop region of human α4 subunits were generated by immunizing rabbits and affinity-purifying the sera as described previously. ^3^

SDS-PAGE, Western blot and fluorescence detection: HEK cells from one 10 cm petri dish were resuspended in 400 µl reducing sample buffer (62.5 mM Tris/HCl pH = 6.8; 5 % α-mercaptoethanol, 2 % SDS; 10 % glycerol, and 0.01 % PyroninY), denatured for 15 min at 65° C and separated on 10% SDS gels using a Tris-glycine buffer system (25 mM Tris, 192 mM glycine, 0.1 % SDS). The size of proteins was determined by mixing 0,017 µl Super Signal Enhanced Molecular Weight Protein Ladder (Thermo Scientific) diluted in 17 µl reducing sample buffer (as described above) with 3 µl SeeBlue Plus2 Pre-Stained Protein Standard (Thermo Scientific).

Gels were blotted semi-dry on pre-wetted polyvinylidene fluoride membranes (Immobilon-P PVDF Transfer Membrane, Millipore) using the TransBlot Turbo Transfer System (Bio-Rad). After blocking with blocking buffer (5 % nonfat dry milk powder in PBS, 0.1 % Tween 20) for 1 hour at room temperature, the membranes were incubated with primary antibody (a mixture of 0.5 µg/ml polyclonal anti-α4 antibody and monoclonal anti-actin-antibody, clone C4 (Millipore MAB1501, 1:5000) in blocking buffer over night at 4° C. Membranes were then washed extensively with washing buffer (1.5 % nonfat dry milk powder in PBS including 0.1 % Tween 20) and incubated for 1 hour at room temperature with a combination of peroxidase-conjugated mouse anti-rabbit light chain-specific and peroxidase-conjugated goat anti-mouse light chain-specific secondary antibodies (both from Jackson ImmunoResearch), each diluted 1:1000 in washing buffer. Following another extensive washing step, membranes were submerged in Immobilon Western Chemiluminescent HRP substrate (Millipore WBKLS0500) for 5 minutes and documented with a Fluor-S Max Multi-Imager (BioRad). A representative experiment is shown in Figure S1.

Quantification: Band intensities were analyzed with the Image Lab software version 5.1 for Windows (Bio-Rad Laboratories, Inc). The actin-signal at 43 kD was used as a loading control to calculate a lane normalization factor. The lane with the highest actin-signal value was identified and the actin signals observed in all other lanes were divided by this signal. Consequently, the signals observed with the anti-α4 antibodies were divided by the corresponding lane normalization factor. Normalized protein expression levels of mutant α4-subunits were compared to normalized levels of WT α4-subunits and analyzed statistically using a one sample t-test in GraphPad Prism version 8.3.0 for Mac OS X, GraphPad Software, La Jolla, California, USA, www.graphpad.com. We found no statistically significant differences between the subunit expression levels of WT and mutated α4-subunits neither in α4β2 nor in α4β3 transfected HEK-293 cells.

**Representative Western Blot**


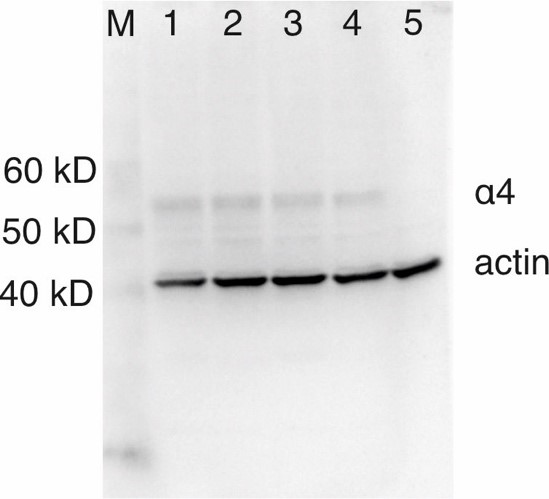


**Figure S1**: Representative Western Blot from three biological replicates of HEK293 cells transfected with α4β2 (Lane 1), α4T300Iβ2 (Lane 2), α4β3 (Lane 3), α4T300Iβ3 (Lane 4) and non-transfected cells (Lane 5). M: Super Signal Enhanced Molecular Weight Protein Ladder (Thermo Scientific)

**Functional Testing with Two Electrode Voltage Clamp (TEVC) in Xenopus laevis oocytes**

Oocytes were bought from Ecocyte Bioscience (grade 1). Delivered oocytes came ready to inject without a follicular layer and were stored at 4°C until use.

Preperation of constructs: All used cDNAs of GABA_A_ receptor subunits were provided by Prof. Sieghart and verified by sequencing, with exception of the human β2 and β3 subunits, which were purchased in the PUNIV vector and sequenced from Eurofins Genomics. The human α4 cDNA was subcloned from the PCI vector into PUNIV by digesting both vectors with NheI and EcoRI fast digestion enzymes from Thermo Scientific and subsequent ligation. For generating the α4T300I mutation the α4-PUNIV vector as template and the primers FW: 5´-AACTGTCCTCACCATGATCACACTAAGCAT, REV: 5´- CACTGATGCTTAGTGTGATCATGGTGAGGA were used, resulting in a substitution of amino acid T300 (ACC) to I (ATC) by using the Q5 Site-Directed Mutagenesis Kit (New England Biolabs) following manufacturer’s instruction. The mutated α4 subunit was confirmed by sequencing.

mRNA Transcription: cDNA Vectors encoding for the rat and human GABA_A_ receptor subtypes were linearized with appropriate endonucleases. Subsequently, the cDNA was purified and concentrated with the DNA Clean and Concentrator TM Kit (Zymoresearch, Catalog No. D4005), transcribed and purified in order to generate mRNA. Capped transcripts of the purified cDNA were produced using the mMESSAGE mMACHINE® T7 transcription kit (Ambion, TX, USA) and polyadenylated using the Ambion PolyA tailing kit (Ambion). After transcription and polyadenylation the RNA was purified with the MEGAclear TM Kit (Ambion, Catalog No. AM1908). The final RNA concentration was measured on NanoDrop® ND-1000 and finally diluted and stored in diethylpyrocarbonate-treated water at −80 °C

RNA mixture: In pilot experiments human subunit combinations (α4β2, α4T300Iβ2, α4β3 and α4T300Iβ3) were mixed in a 1:1; 5:1 and 10:1 ratio. Since mixtures of 1:1 and 5:1 did not result in measureable currents, all further steps were done with a 10:1 mixture of α4/4T300Iβ2/3 subunits.

Rat binary subunit combinations (α4β3, α4T300Iβ3) were mixed in a 1:1 ratio, since this established ratio produces microampere currents after 3-4 days of incubation. Tertiary combinations including the delta subunit (α4β3δ and α4T300Iβ3δ), were mixed in a 1:1:5 ratio, as described previously.^4^

Injection of *Xenopus Laevis* oocytes: All final RNA mixtures had a concentration of 70 ng/µl. Oocytes were injected with the Drummond Nanoject II (Drummond Scientific Company) using a thin pulled (Sutter Instruments Model P-97) glas microcapillary with 46nl of RNA for a total amount of 3,22 ng.

Incubation of oocytes: After injection, oocytes were kept in Ca^2+^ free ND-96 buffer for 1 hour at 18°C in order to preselect and dispose of damaged cells.

The oocytes injected with human subunit RNA were incubated (NDE++) at 23°C for 9-10 days before recording. Oocytes injected with the rat subunit RNA were incubated (NDE++) at 18°C for 3-4 days before recording.

Buffer compositions:

Buffer for injection (ND-96): (96mM NaCl, 2mM KCl, 1mM MgCl_2_x6H_2_O; 5mM HEPES)

Incubation buffer (NDE++): (96mM NaCl, 2mM KCl, 1mM MgCl_2_x6H_2_O; 5mM HEPES, 2.5mM pyruvate, 10.000 Units/ml penicilline, 10mg/ml streptomycine), pH adjusted to 7.5 with NaOH, steril filtered.

Measurement/perfusion buffer (NDE): (96mM NaCl, 2mM KCl, 1mM MgClx6H_2_O; 5mM HEPES, 1,8mM CaCl_2_), pH adjusted to 7.5 with NaOH, steril filtered)

Electrophysiological recordings with two electrode voltage clamp

Oocytes were placed on a nylon grid, in a bath of Ca^2+-^containing NDE solution. For current measurements the oocytes were impaled with two microelectrodes (1–3 MΩ resistance) filled with 2 M KCl and continuously perfused with a saline solution that could be switched with a valve to NDE containing GABA and/or THDOC. Oocytes were clamped at -60mV after perfusion started. GABA powder was weighed and freshly diluted in NDE buffer before usage. THDOC was freshly diluted in NDE before usage from a DMSO-stock solution resulting in a final DMSO-concentration of 0.1-0.2 %. All compounds were applied for 30 seconds. Between two applications, oocytes were washed with NDE buffer for up to 20 minutes to ensure full recovery from desensitization before the next application. For dose responses, increasing concentrations of GABA were applied to the cell. All recordings were performed at room temperature with a Turbo Tec-03X npi amplifier. Data were digitized, recorded and measured using an Axon Digidata- 1550 low-noise data acquisition system (Axon Instruments, Union City, CA). Data acquisition was done using pCLAMP v.10.5 (Molecular Devices™, Sunnyvale, CA).

Statistical analysis for *Xenopus laevis* oocytes experiments: Each experiment was conducted in at least 2 batches of oocytes. For each experiment 5 or more biological replicates were collected and exactly specified in the respective figure legends for each experimental condition.

Data were analysed using GraphPad Prism v.8. and plotted as concentration-response curves, averaged traces and bar graphs. The dose response curves were normalized and fitted by non-linear regression analysis to the equation Y = bottom + (top-bottom)/1+10^(LogEC50-X)*^*^n^*^H^, where EC_50_ is the concentration of GABA that elicits 50% of the maximal current, and nH is the Hill coefficient. THDOC modulation of GABA elicited current was defined as (I_GABA+Comp_/I_GABA_), where I_GABA+Comp_ is the current response in the presence of GABA and THDOC and I_GABA_is the control GABA current. Extent of desensitization as a measure of channel inactivation is defined as 1-I(_res_)/I(_peak_), where I_res_ is the remaining current when GABA application ends and I_peak_ is the maximal current. Bar graphs were generated by a calculating means with SEM for each experimental condition. Normalized and averaged traces were generated by normalizing each trace to its peak value (100%). Subsequently, individual traces were aligned for the rising phase and means with SEM calculation was performed for each time point. Means were plotted as black traces and SEM as coloured area. Note that the averaged traces might have a peak slightly different from 100% if individual traces have a difference in rising time.

For all comparisons between WT and mutant receptors, a multiple t-test (unpaired, p<0.05) with Holm-Sidak correction was performed.

**Mean peak current amplitudes**

**Table S1***:* Table providing the unpaired multiple t-test with Holm-Sidak correction results for the difference of mean current amplitudes of binary α4 receptors and their mutated counterparts. As indicated by the p-values, the α4 mutation causes a trend for higher currents, which does not reach statistical significance for any GABA concentration given.

**GABA dose responses recorded from oocytes expressing human subunit combinations**


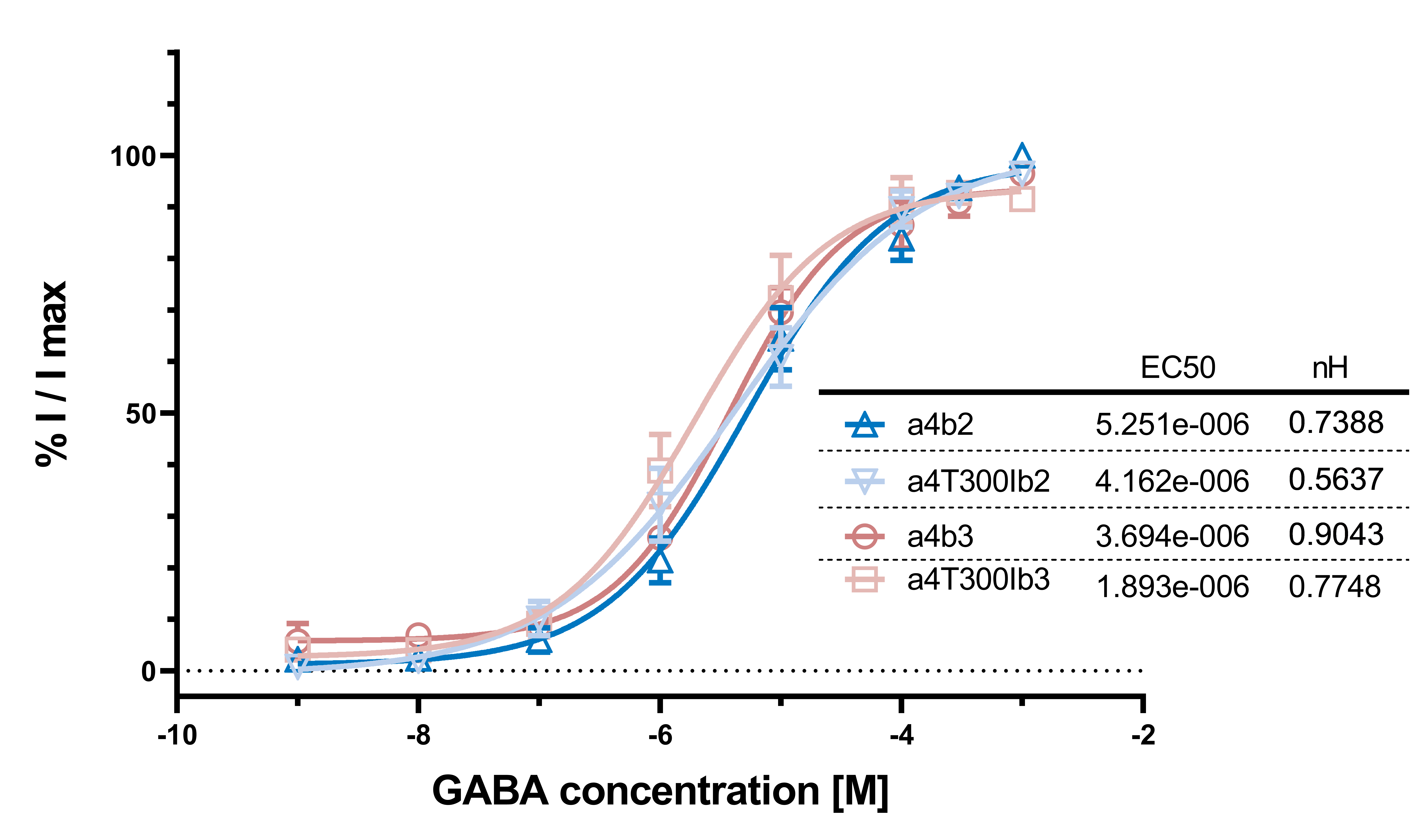


**Figure S2**: Normalized dose response curves of GABA elicited peak currents. The respective subtypes are coloured as indicated in the legend (α4β2: dark blue; α4T300Iβ2: light blue; α4β3: dark red; α4T300Iβ3 light red). All points are given as mean (symbols) ±SEM for each subtype.; α4β2: n=6-7; α4T300Iβ2:n=10-12; α4β3: n=12; α4T300Iβ3:n= 9-10; EC50 values in [M] and Hill slopes are shown for the respective receptor subtypes.

**Supporting data obtained with rat subunits:**

The α4 subunit of rats differs only in a few amino acid positions from the human one, and the pore forming transmembrane domain is completely conserved, see alignment image in supplementary Figure S3:

**
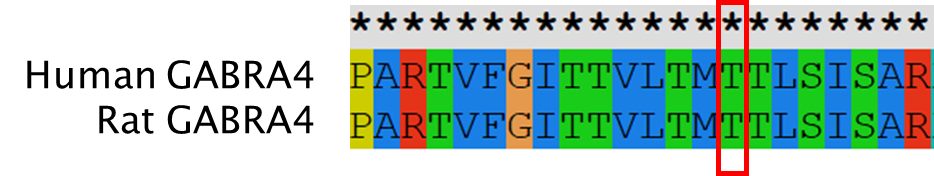
**

**Figure S3:** ClustalX- alignment of human and rat α4 subunits (obtained from UniProt) in the pore forming TM2, where the patient mutation is located. Amino acids are given in a single letter code. Each amino acid has a default color coding assigned from ClustalX (blue=hydrophobic; red=positive charge; green=polar; yellow=prolines; orange= glycines). Star annotation above indicates identical amino acids. The red box indicates the mutated position in α4T300I. Our constructs match the canonical sequences at UniProt.

In spite of the high sequence similarity, the human α4- subunit, when expressed in combination with beta subunits, is known to express poorly (as evidenced by the long expression at elevated temperature, see supplementary methods), and the resulting currents are low.^5^ As a result, on average only one out of 25-30 oocytes injected with human α4- subunit containing combinations can be used for data collection. In contrast, the yield is almost 100% with the corresponding rat subunit combination injected oocytes, in our hands. This is mainly due to the much shorter expression time, and the satisfactory expression at the lower temperatures that are best for the *X.l.* oocytes.

Thus, we took advantage of the robust and easy handling of the rat subunits and collected a large dataset to further support the observed changes in desensitization kinetic induced by the mutation in the TM2.

First, we compared the behaviour of α4β3 and α4T300Iβ3 receptors formed by rat subunits to the human homologs, with similar results regarding receptor kinetics (Figure S4). We then included additionally the α4β3δ subunit combination. α4βδ containing receptors are discussed in the literature as a receptor subtype that provides tonic, slow desensitizing currents and were shown to be involved in pathomechanisms of seizures.^6^ Specifically, a recent study demonstrated in a panel of 10 patients with epileptic and or neurodevelopmental abnormalities with 7 different mutations in the delta subunit, that α4βδ receptors display molecular changes consistent with aberrant tonic inhibition.^6^

The comparison in desensitization between α4β3δ and α4T300Iβ3δ shown in Figure S4 is further confirming a dominant effect of the mutation in the α4 subunit. In total, the datasets collected with the rat subunit combinations demonstrate that the accelerated desensitization is observed reproducibly and robustly in a large number of cells and at peak current levels that are higher compared to those reached with the human subunits, strongly supporting this as a property induced by the mutation.

**Extent of desensitization, rat subunit combinations**





**Figure S4:** Quantitative comparison of the extent of desensitization in rat subunit combinations of α4β3 (dark red), α4T300Iβ3 (light red), α4β3δ (dark brown) and α4T300Iβ3δ (light brown). Data is shown as mean (bars) ± SEM with each data point representing a biological replicate for the respective subtype and GABA concentration. α4β3: n(100nM; 1µM; 10µM; 100µM; 300µM; 1000µM)= 10; 13; 13; 13; 13; 12. α4T300Iβ3: n(100nM, 1µM; 10µM; 100µM; 300µM; 1000µM)= 9. α4β3δ: n(100nM, 1µM; 10µM; 100µM; 300µM; 1000µM)= 8, 9, 9, 9, 9, 9. α4T300Iβ3δ: n(100nM, 1µM; 10µM; 100µM; 300µM; 1000µM)=9; 8; 9; 9; 9; 9. p values: **P=0.009 ***P<0.001; multiple t-tests with Holm-Sidak correction.

**Neurosteroid interaction sites in the TMD**

Neurosteroids are an important class of endogenous molecules, produced by neurons and glia cells^7,8^ in the central nervous system (CNS), with a wide range of functions. Preclinical evidence in mouse models points towards a critical involvement of neurosteroids in controlling seizures.^9-11^ As a consequence, they are currently being developed as anticonvulsants, but also as sedatives and antidepressants. The first therapeutic neurosteroid for the treatment of postpartum depression on the market is the endogenous molecule allopregnanolone (also called brexanolone).^12^ Among their main targets in the CNS are GABA_A_ receptors, at which they are potent modulators. In the past, the attempt to identify neurosteroid binding sites on GABA_A_ receptors was fruitful, with multiple methodologies converging in overlapping residues of importance for their action (Figure S5). Besides the canonical binding site at the β+/α- interface of the transmembrane domain in which THDOC and other neuroactive steroids were found,^13^ an α-intra and a β- intra subunit binding pocket were proposed based on photolabelling studies.^14^ The mutation α4T300I is located at the same protein domain (TMD), as the neurosteroid binding sites (Figure S5). Additionally, very recently neurosteroids were shown to modulate GABA effects in a site and activation dependent manner,^15^ which is why a change of receptor kinetics due to the mutation might contribute to the THDOC loss of function in the mutated a4β receptors we observe.


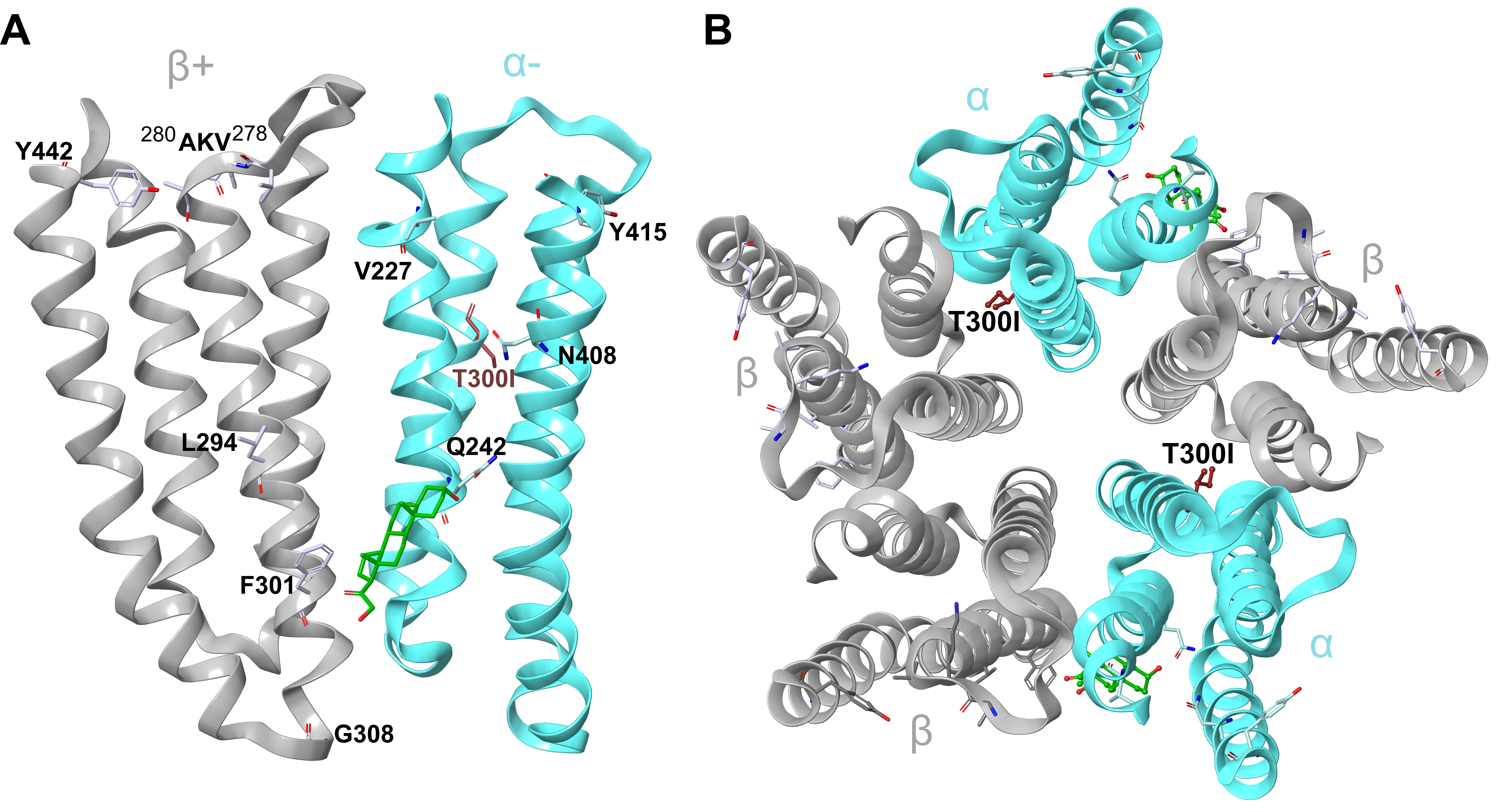


***Figure S5***: GABA_A_ receptor transmembrane domain (TMD) homology model with generally assumed arrangement on the basis of 5OSB: Left: β+/α- interface from the side view with THDOC (green) bound; right: transmembrane domain of a GABA_A_ receptor pentamer from top view with two α4 (cyan) and three β (grey) subunits. All residues which might be relevant for neurosteroid interactions based on the study by Chen et al are labelled.

**Supplementary References**

1. Simeone X, Koniuszewski F, Müllegger M, Smetka A, Steudle F, Puthenkalam R, et al. A benzodiazepine ligand with improved gabaa receptor a5-subunit selectivity driven by interactions with loop C. Mol Pharmacol . 2021 Jan 1;99(1):39–48.

2. David R, Ciuraszkiewicz A, Simeone X, Orr-Urtreger A, Papke RL, McIntosh JM, et al. Biochemical and functional properties of distinct nicotinic acetylcholine receptors in the superior cervical ganglion of mice with targeted deletions of nAChR subunit genes. Eur J Neurosci [Internet]. 2010 Mar;31(6):978–93.

3. Mossier B, Togel M, Fuchs K, Sieghart W. Immunoaffinity purification of γ-aminobutyric acid(A) (GABA(A)) receptors containing γ1-subunits. Evidence for the presence of a single type of γ- subunit in GABA(A) receptors. J Biol Chem. 1994 Oct 14;269(41):25777–82.

4. Lee HJ, Absalom NL, Hanrahan JR, van Nieuwenhuijzen P, Ahring PK, Chebib M. A pharmacological characterization of GABA, THIP and DS2 at binary α4β3 and β3δ receptors: GABA activates β3δ receptors via the β3(+)δ(−) interface. Brain Res. 2016;1644:222–30.

5. Wang M De, Rahman M, Johansson IM, Bäckström T. Agonist function of the recombinant α4β 3δ GABAA receptor is dependent on the human and rat variants of the α4-subunit. Clin Exp Pharmacol Physiol.2010; 37(7):662–9.

6. Ahring PK, Liao VWY, Gardella E, Johannesen KM, Krey I, Selmer KK, et al. Gain-of-function variants in GABRD reveal a novel pathway for neurodevelopmental disorders and epilepsy. Brain. 2021 Oct 11

7. Agís-Balboa RC, Pinna G, Zhubi A, Maloku E, Veldic M, Costa E, et al. Characterization of brain neurons that express enzymes mediating neurosteroid biosynthesis. Proc Natl Acad Sci. 2006 Sep 26;103(39):14602–7.

8. Faroni A, Magnaghi V. The neurosteroid allopregnanolone modulates specific functions in central and peripheral glial cells [Internet]. Vol. 2, Frontiers in Endocrinology. Front Endocrinol (Lausanne); 2011.

9. Miziak B, Chrościńska-Krawczyk M, Czuczwar SJ. Neurosteroids and Seizure Activity [Internet]. Vol. 11, Frontiers in Endocrinology. Frontiers Media S.A.; 2020.

10. Gasior M, Ungard JT, Beekman M, Carter RB, Witkin JM. Acute and chronic effects of the synthetic neuroactive steroid, ganaxolone, against the convulsive and lethal effects of pentylenetetrazol in seizure-kindled mice: comparison with diazepam and valproate. Neuropharmacology. 2000 Jun 1;39(7):1184–96.

11. Kaminski RM, Livingood MR, Rogawski MA. Allopregnanolone analogs that positively modulate GABAA receptors protect against partial seizures induced by 6-Hz electrical stimulation in mice. Epilepsia. 2004 Jul;45(7):864–7.

12. Kanes S, Colquhoun H, Gunduz-Bruce H, Raines S, Arnold R, Schacterle A, et al. Brexanolone (SAGE-547 injection) in post-partum depression: a randomised controlled trial. Lancet. 2017 Jul 29;390(10093):480–9.

13. Laverty D, Thomas P, Field M, Andersen OJ, Gold MG, Biggin PC, et al. Crystal structures of a GABA A -receptor chimera reveal new endogenous neurosteroid-binding sites. Nat Struct Mol Biol. 2017 Oct 2;24(11):977–85.

14. Chen ZW, Bracamontes JR, Budelier MM, Germann AL, Shin DJ, Kathiresan K, et al. Multiple functional neurosteroid binding sites on gabaa receptors. Khosla C, editor. PLoS Biol. 2019 Mar 7;17(3):e3000157

15. Sugasawa Y, Cheng WWL, Bracamontes JR, Chen ZW, Wang L, Germann AL, et al. Site-specific effects of neurosteroids on gabaa receptor activation and desensitization. Elife. 2020 Sep 1;9:1–32.
